# Intranasal Dexmedetomidine on Stress Hormones, Inflammatory Markers, and Postoperative Analgesia after Functional Endoscopic Sinus Surgery

**DOI:** 10.1155/2015/939431

**Published:** 2015-06-25

**Authors:** Chaoliang Tang, Xiang Huang, Fang Kang, Xiaoqing Chai, Song Wang, Guobing Yin, Hongtao Wang, Juan Li

**Affiliations:** Department of Anesthesiology, Anhui Provincial Hospital, Anhui Medical University, Hefei, China

## Abstract

*Background*. A strong ongoing intraoperative stress response can cause serious adverse reactions and affect the postoperative outcome. This study evaluated the effect of intranasally administered dexmedetomidine (DEX) in combination with local anesthesia (LA) on the relief of stress and the inflammatory response during functional endoscopic sinus surgery (FESS).* Methods*. Sixty patients undergoing FESS were randomly allocated to receive either intranasal DEX (DEX group) or intranasal saline (Placebo group) 1 h before surgery. Stress hormones, inflammatory markers, postoperative pain relief, hemodynamic variables, blood loss, surgical field quality, body movements, and satisfaction were assessed.* Results*. Plasma epinephrine, norepinephrine, and blood glucose levels were significantly lower in DEX group as were the plasma IL-6 and TNF-*α* levels (*P* < 0.05). The weighted areas under the curve (AUCw) of the VAS scores were also significantly lower in DEX group at 2–12 h after surgery (*P* < 0.001). Furthermore, hemodynamic variables, blood loss, body movements, discomfort with hemostatic stuffing, surgical field quality, and satisfaction scores of patients and surgeons were significantly better (*P* < 0.05) in DEX group.* Conclusions*. Patients receiving intranasal DEX with LA for FESS exhibited less perioperative stress and inflammatory response as well as better postoperative comfort with hemostatic stuffing and analgesia.

## 1. Introduction

Dexmedetomidine (DEX) is a highly selective *α*2 receptor agonist with sedation, analgesia, and anxiolytic in clinical practice [1, 2]. It has an analgesic-sparing effect, significantly reducing opioid requirements in both intraoperative and postoperative period [3].

DEX also has a sympatholytic effect, which can reduce the stress response to surgery and ensure a stable hemodynamic state [4]. Few of clinical studies have explored the use of intravenous DEX in attenuation of stress and inflammatory response and postoperative pain. In a pediatric cardiac surgery study, DEX at a 1 *μ*g/kg loading dose followed by infusion at 0.5 *μ*g/kg/h until separation from cardiopulmonary bypass, which was effective in blunting stress and inflammatory response to surgical trauma as indicated by lower norepinephrine level, IL-6, and IL-10 ratio, while achieving better postoperative pain control [5]. In another pediatric cardiac surgery study using caudal bupivacaine 0.25%, 2.5 mg/kg and DEX 0.5 *μ*g/kg attenuated stress response to surgical trauma and provided better postoperative analgesia [6]. In view of these consistent clinical effects, future studies with different routes of administration have been suggested.

Intranasal DEX is convenient, effective, and noninvasive and also has useful analgesic and sedative effects in surgical procedures [7]. Cheung's research has shown that intranasal DEX 1 and 1.5 *μ*g/kg in surgical procedures produced significant sedation and less postoperative pain [7, 8].

However, the clinical use of intranasal DEX for the stress and inflammatory response relief affection in surgical procedures has not been explored. We therefore conducted this prospective, randomized, and double blind study to explore the efficacy of intranasal DEX in regard to operative conditions, the stress and inflammatory response relief, and postoperative analgesia following FESS.

## 2. Materials and Methods

This prospective, randomized, and double blind clinical trial was approved by the Ethics Committee of Anhui Provincial Hospital, Anhui Medical University, and registered at Chinese Clinical Trial Registry (ChiCTR) with registration number ChiCTR-TRC-14004886. Informed consent was obtained from all the patients. Patients of either sex with the American Society Anesthesiologists physical status I-II, aged between 18 and 60 years, undergoing FESS under LA were recruited.

Exclusion criteria were pregnancy, history of serious adverse reaction, or patients with known sensitivity to local anesthetic drug and allergy to study drugs, patients with BMI >35 kg/m^2^, ischemic heart disease, asthma, sleep apnoea syndrome, use of *α*2-agonists or–antagonists, hypotension (baseline systolic arterial pressure (SAP), 100 mmHg), and bradycardia (baseline heart rate (HR), 60 beats/min), patients on pain perception modifying drugs, and those with history usage of any opioid or sedative medications in the week prior to surgery.

The Visual Analogue Scale (VAS) (0, no discomfort and no pain; 10, a high level of discomfort and maximum pain) was explained to the patients during the preoperative visit. The patients were randomized to the two study groups by random number table method, which was prepared by an unwitting statistician, Placebo group, and DEX group (*n* = 30).

All patients were sent to the anesthesia preoperative room without any premedication 60 min before the surgery. Standard monitoring consisted of five-lead electrocardiography (ECG), oxygen saturation (SpO_2_), and noninvasive blood pressure. One anesthesiologist who was unaware of the clinical nature of the study monitored and conducted the case. Study drug, either undiluted DEX 1.5 *μ*g/kg (DEX group) or the same volume of 0.9% normal saline (Placebo group), was administered to each naris as drops. Patients did not access the operating room until surgery. One anesthesia nurse who was not involved in the experiment prepared all the study drugs. The anesthesiologist in the operating room who was blinded to group assignment collected the date.

One hour after study drug administration, patients were then transferred to the operating room for the surgical procedure. An infusion of Ringer's solution 8~10 mL/kg was given after a 20-gauge intravenous cannula was inserted in the dorsum of each patient's left hand. No additional sedative premedication was used. All the operations were done by the same surgical team of three otolaryngology surgeons, who also performed the LA with the standard FESS surgical technique in both patient study groups before the surgical procedure that involves submucosal infiltrative anesthesia with the 2 mL mixture of 1% lidocaine and epinephrine (1 : 100,000) in the region of the agger nasi cell and the middle turbinate; then packed nasal passages and total nasal passages with cotton pieces contain 1% tetracaine 20 mL and 1‰ adrenaline 8 mL under nasal endoscope. After confirming adequate analgesia, the surgical procedure was then commenced.

Systolic blood pressure (SBP), diastolic blood pressure (DBP), HR, and SPO_2_ were recorded at seven time points (T0, baseline; T1, 5 min after intranasal instillation; T2, 30 min after intranasal instillation; T3, before local anesthesia; T4, the beginning of the operation; T5, 30 min after operation; T6, the end of operation; T7, out of PACU). After the procedure, patients were transferred to the PACU and hemodynamic parameters; degree of analgesia was monitored until transferred to surgical ward. Patients' body movements leading a temporary stop to surgical procedure, caused by pain or discomfort during procedure, were also recorded. Blood loss was documented. Quality of intraoperative surgical field during FESS was evaluated by the surgeons with Formmer's scores of surgical field quality (1, bleeding, so mild it was not even a surgical nuisance; 2, moderate bleeding, a nuisance but without interference; 3, moderate bleeding that moderately compromised surgical; 4, bleeding, heavy but controllable, that significantly interfered; 5, massive uncontrollable bleeding) [9]. The degree of adverse reactions of hemostatic stuffing after FESS was also evaluated (1, no swelling, can tolerate; 2, swelling, can barely tolerate; 3, swelling, cannot tolerate). Surgeons who were asked to evaluate their overall satisfaction with the procedure (immediately after the surgery), and patients who were also asked to evaluate their overall satisfaction with the procedure (when shifted to surgical ward and 48 h after the procedure) by numerical rating scale (NRS; 0, being least satisfied; 10, being most satisfied). The standard VAS score was used to evaluate postoperative pain at 2 h, 4 h, 8 h, 12 h, 24 h, and 48 h postoperatively.

Three milliliter of venous blood was collected on the morning of the operation day and arrival in the PACU. One drop of blood was taken to measure blood glucose level (GLU). The rest was added to tubes without anticoagulant, perfectly still until the serum separation, the serum precipitated was taken with centrifugal to centrifuge at 4000 rpm in 4°C for 10 min, and then the supernatant was sucked out to place in −80°C cryogenic refrigerator to wait to test epinephrine, norepinephrine, interleukin-6 (IL-6), interleukin-8 (IL-8), and tumor necrosis factor-alpha (TNF-*α*). Epinephrine and norepinephrine were assayed by enzyme-linked immunosorbent assay (ELISA) (2-CAT; Elabscience Biotechnology Co., Ltd). IL-6, IL-8, and TNF-*α* was measured with the Immulite Automated Chemiluminometer (Siemens Healthcare Diagnostics, Deerfield, IL).

Our primary outcome measures were stress hormones and inflammatory markers levels; the secondary outcomes were postoperative amplification of pain sensation and discomfort with hemostatic stuffing. The sample size estimation was based according to norepinephrine, the most intuitionistic one of the stress hormones. A pilot study of 10 patients at our center found that the mean ± standard deviation (SD) of norepinephrine for FESS was 466.94 ± 73.74. A reduction of 10% in norepinephrine (420.25 ± 66.37) after intranasal DEX in the treatment group was considered clinically significant, and this required a sample size of 29 per group to achieve a power of 80% and a type I error of 5%. To compensate for the possibility of dropout, we recruited 60 patients, 30 patients per group. The VAS pain scores over the 48 postoperative hours are expressed as AUC using the trapezoid rule and were analyzed by the Mann-Whitney *U* test. The demographic characteristics data were evaluated using unpaired *t*-test for between-group and paired *t*-test for within group comparisons. The *χ* ^2^ test was used to analyze categorical variables. Student's *I* test and ANOVA were used for unpaired quantitative variables. Data are presented as Mean ± SD (SEM), count (%), or as Median (IQR (range)). *P* value less than 0.05 was considered as significant. The statistical analyses were performed using SPSS Statistics 13.0 software.

## 3. Results

**Figure 1:**
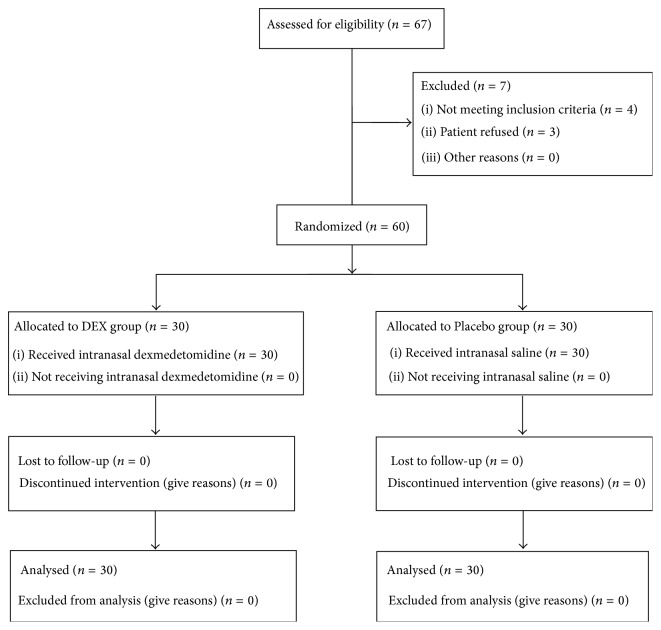
Flow diagram of patient recruitment.

Sixty patients were recruited from August 2014 to February 2015. No assigned patients withdrew from the study (Figure 1). All patients underwent their planned surgical procedure and received their allotted study drug.

Table 1 shows the patient and procedural characteristics of both groups, respectively. No significant difference was seen in demographic data, surgical characteristics, and duration of operation between the two study groups. However, intranasal DEX resulted in a significant reduction in blood loss (*P* = 0.030).

**Figure 2:**
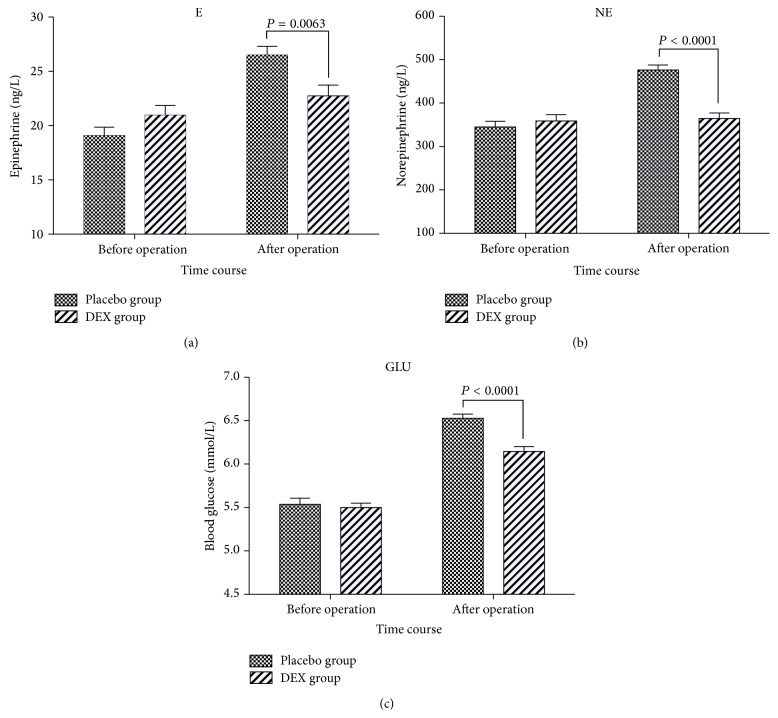
Plasma epinephrine, norepinephrine, and blood glucose level of patients receiving intranasal DEX and placebo before operation and after operation. Values are given as mean ± SEM. Between-group comparison of epinephrine, norepinephrine, and blood glucose after operation. DEX group was lower than that of Placebo group (*P* = 0.0063, *P* < 0.0001, and *P* < 0.0001, resp.).

Plasma concentrations of epinephrine, norepinephrine, and blood glucose before operation and after operation are displayed in Figure 2. All were with no difference between the groups before operation and they all increased in both groups after operation compared to before operation, but the difference of the three variables was significant in Placebo group in contrast with only the blood glucose in DEX group (*P* < 0.0001). Between-group comparison of epinephrine, norepinephrine, and blood glucose after operation and DEX group was lower than that of Placebo group (at *P* = 0.0063, *P* < 0.0001, and *P* < 0.0001, resp.).

**Figure 3:**
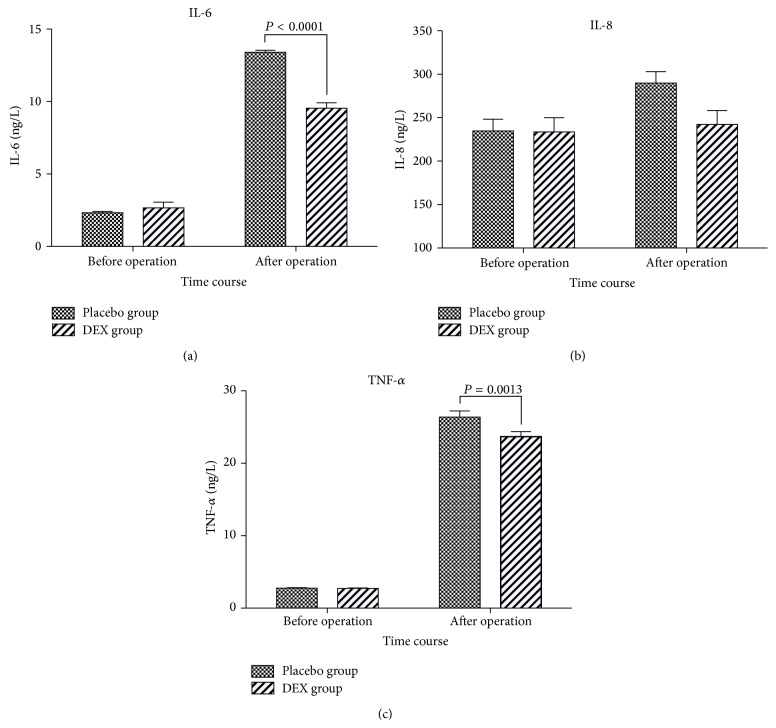
Plasma IL-6, IL-8, and TNF-*α* concentration of patients receiving intranasal DEX and placebo before operation and after operation. Values are given as Mean ± SEM. IL-6 and TNF-*α* were significant lower in DEX group after operation (*P* < 0.0001 and *P* = 0.0013, resp.).

Plasma concentrations of IL-6, IL-8, and TNF-*α* were with no difference between the groups during preoperation and they all increased in both groups after operation compared to before operation, and the difference of IL-6 and TNF-*α* was significant in contrast with no difference about IL-8 in both groups. IL-6 and TNF-*α* were significantly lower in DEX group after operation (at *P* < 0.0001 and *P* = 0.0013, resp.) (Figure 3). IL-8 was also lower in DEX group; however the difference was no significant.

The degree of adverse reactions of hemostatic stuffing was shown in Table 2. More patients from DEX group than Placebo group could tolerate the hemostatic stuffing (*P* = 0.031).

**Figure 4:**
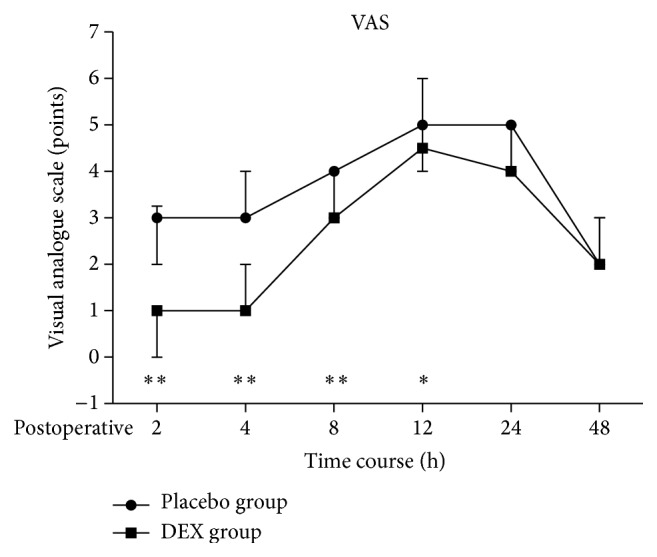
Median postoperative VAS pain scores of patients receiving intranasal DEX and placebo at each recording time point. The areas under curves (AUC) of VAS pain scores for 2–12 h were significantly lower in DEX group than Placebo group (*P* < 0.05). Values are given as Median (IQR (range)). ^*∗*^ *P* < 0.001, ^*∗∗*^ *P* < 0.0001.

Mean AUC of VAS pain scores for postoperative 2–12 h were 26.2 and 39.4 for patients in groups DEX and placebo, respectively (*P* < 0.001); while mean AUC of VAS pain scores for postoperative 12–48 h were 139.2 and 145.4 for patients in groups DEX and placebo, respectively (*P* = 0.741) (Figure 4). Although the AUC of postoperative VAS pain scores at 2–12 h was significantly lower in DEX group, no similar pain relief was noticed for postoperative AUC VAS at 12–48 h.

**Figure 5:**
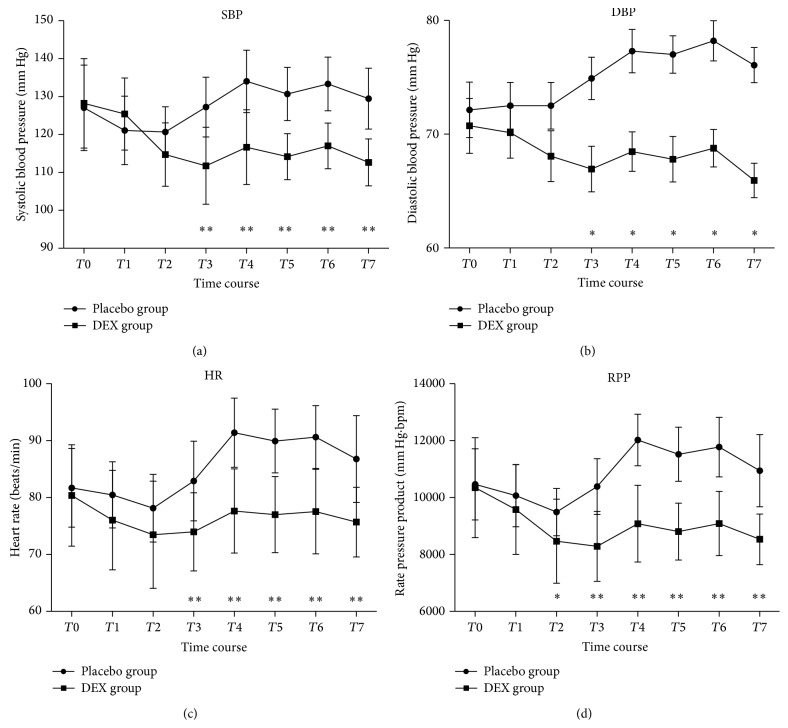
Hemodynamic variables of patients receiving intranasal DEX and placebo at each recording time point. Values are given as Mean ± SD. ^*∗*^ *P* < 0.05; ^*∗∗*^ *P* < 0.0001. Baseline (T0), 5, 30 min after intranasal instillation (T1, 2), before local anesthesia (T3), the beginning of the operation (T4), 30 min after operation (T5), the end of operation (T6), and out of PACU (T7). Between-group comparison of hemodynamic variables at the same time period after intranasal drugs (from T3 to T7); DEX group was lower compared to Placebo group (*P* < 0.05, *P* < 0.0001, resp.).

There was no difference about SAP, DBP, HR, and Rate Pressure Product (RPP) at baseline between the two groups, and they both had a rise over time during the operation (from T3 to T6). However, the difference about HR and RPP was significant in Placebo group (*P* < 0.0001). Between-group comparison of hemodynamic variables at the same time period after intranasal drugs (from T3 to T7) and DEX group was lower compared to Placebo group (at *P* < 0.05 and *P* < 0.0001, resp.) (Figure 5).

Concerning the surgical field quality, more surgeons from DEX group than Placebo group were satisfied (*P* = 0.046) (Table 3). Due to pain or discomfort, more patients from Placebo group than DEX group had body movements that affected the normal operation (*P* = 0.035) (Table 4). When asked about satisfaction with the procedure, both surgeons and patients from DEX group felt better and would use the same drug again (at *P* < 0.001 and *P* < 0.001, resp.) (Table 5).

## 4. Discussion

This randomized, double-blinded, and comparative study was undertaken to evaluate the use of intranasal DEX in FESS. Our principal findings were that patients given intranasal DEX during FESS were safe and effective and experienced significantly slighter hemodynamic changes and perioperative stress and inflammatory response, less postoperative discomfort, and pain in the early postoperative period, and the surgeons also had better surgical field and more satisfaction with its use.

FESS is usually performed under local anesthesia, which is always interrupted because of the fear, anxiety, pain, discomfort, and cardiovascular stress of patients, and the complications such as bleeding and postoperative pain are always existent [10]. Excessive and prolonged intraoperative stress may cause severe adverse reaction and influence postoperative outcome [11]. The release of inflammatory mediators caused by surgical trauma may directly cause pain [12]. The modification of stress and inflammatory response may potentially be useful in attenuating the postoperative pain and discomfort and improve the postoperative outcome [13]. Recently, monitored anesthesia care (MAC) and general anesthesia (GA) are rapidly used but they both have their defects such as the risk of respiratory depression and the high medical costs [14, 15]. Considering the hemorrhagic secretions into the mouth during the process of the surgery, many surgeons do not want to give patients additional anesthesia sedatives; they need the patients' cooperation to spit the hemorrhagic secretions. Anyway, FESS could be done under LA without any sedatives safely and feasibly, despite more or less a bit discomfort [16, 17].

Intravenous DEX in ENT surgeries like functional endoscopic sinus surgery (FESS), middle ear surgeries, thyroplasty, and septoplasty under monitored anesthesia care (MAC) has been reported to be safe and effective [18–21]. Although DEX has both sedative and analgesic properties and has been used as a single agent in many painful procedures, the analgesic potential, however, does not approximate the potency of opioids, many patients who received DEX required supplemental analgesia [22]. However, using DEX in a multimodal manner may be useful to allow lower doses of each component drug and, potentially, side effects similar to the way it is used during general anesthesia [23]. The advantage of antisialagogue effect also could improve the efficacy of local anesthesia.

Changes in hemodynamic properties in response to surgical stress, such as increase in blood pressure, HR, and RPP were significantly lower in DEX group. These decreases may be useful for clinic that significantly reduced the need for vasopressors or anticholinergic support. Our results are similar to other studies which lower HR and blood pressure that were observed in the DEX group [7, 8]. It could be explained by the markedly decreased sympathetic activity [24]. We tested the plasma stress hormones to verify the theory and found that plasma epinephrine, norepinephrine, and blood glucose were indeed significantly lower in DEX group. FESS is a commonly performed procedure; however, close proximity of the surgical field to major blood vessels makes the surgical field associated with blood loss easy [25]. The stable and lower hemodynamics made the surgical field associated with less blood loss in DEX group. In view of this, surgeons could do the operation better and faster, and most of surgeons from the DEX group were satisfied with its use. These findings suggest that the combined effect of intranasal DEX and local anesthesia in reducing the response to surgical stress and inducing hypotension may minimize the risk of surgical complications and stabilize hemodynamics for FESS.

Clinical studies have shown that the acute pain caused by FESS could lead patients to sleep deprivation [15, 26]. Also postoperative pain has become one of the most important factors that affect patients' postoperative recovery [27]. Postoperative pain due to FESS should be managed aggressively as the earlier treatment of analgesia, the better the results [27, 28].

Previous studies have suggested that postoperative pain of nasal endoscope is mainly due to the stimulation of the surgery itself. Surgical trauma leads to the release of inflammatory mediators from immune cells and nonneuronal cells in the periphery, directly causes pain [12]. The proinflammatory cytokines (PICs) such as interleukin-6 (IL-6) and tumor necrosis factor-*α* (TNF-*α*) are an important group of inflammatory mediators and play an essential role in pain sensitization [29, 30]. The peripheral effects of these PICs on sensitizing nociceptors have been well documented. On the one hand, part of which is passed to the nerve center of the more advanced, produced reaction on the spinal cord and cortical, which eventually form the pain experience; on the other hand, part of the peripheral nociceptive stimulus can directly lead to central sensitization and super reactivity to cause more severe pain [31]. Therefore, systemic or regional analgesic regimens initiated before the onset of surgery can prevent both peripheral and central sensitization, thereby attenuating the postoperative amplification of pain sensation [27, 31, 32]. Significantly increased IL-6 and TNF-*α* serum levels have been detected in our patients after operation in both groups. DEX group had a lower inflammatory cytokines levels compared to Placebo group after operation. This result is consistent with our findings of postoperative VAS pain scores, which DEX group also had a significantly lower VAS pain scores for postoperative 2–12 h. Therefore, we think that our results could give a suggestion that the beneficial effects of intranasal DEX initiated before the onset of surgery would be effective to reduce the inflammatory response, so as to reduce the postoperative pain sensitization.

As the experience of the FESS of the author himself, the postoperative pain mainly occurs during the postoperative 24 h, the nasal cavity-filling period [33]. The pain could slowly ease or disappear after hemostatic stuffing extraction [34]. Nasal cavity filling is an important means of FESS postoperative hemostasis; however, nasal cavity filling may inevitably have some adverse reactions, such as elevated intraocular pressure, and difficulty in swallowing. Furthermore, long time mouth breathing could cause postoperative sore throat and influence the patient's sleep. Then the patients' mood will be worse and lead to the pain sensitization aggravating. Our research suggests that intranasal DEX initiated before the onset of FESS could significantly reduce the postoperative discomfort.

Not only the surgeons but also the patients in DEX group were generally satisfied with the procedure. More optimal operating conditions, less blood loss, and better cooperation of patients got the surgeons' approval; moderate sedation, slight adverse reaction of hemostatic stuffing, and less postoperative pain also improved the patients' satisfaction.

In conclusion, intranasal DEX with local anesthesia used in FESS could yield more stable hemodynamic profile and lower stress and inflammatory response and attenuate the adverse reactions of hemostatic stuffing and pain sensitization at the early postoperative period, leading to better surgical field and satisfaction with surgeons and patients. Stress-free anesthesia, with measurement-based control of surgery stress, would improve postoperative outcome.

## Figures and Tables

**Table 1:** Subject and procedure characteristics.

Characteristic	Treatment groups		
	Placebo group (*n* = 30)	DEX group (*n* = 30)	*P* value
Age (years)	37.4 (12.5)	37.2 (12.3)	0.951
Gender (M/F)	17/13	14/16	0.747
Weight (kg)	67.1 (8.3)	64.7 (9.6)	0.305
Height (cm)	163.5 (8.3)	163.1 (7.9)	0.867
*Procedures *			
Septoplasty (*n*)	12 (40%)	9 (30%)	NS
Functional endoscopic sinus surgery (*n*)	12 (40%)	15 (50%)	NS
Septoplasty + functional endoscopic sinus surgery (*n*)	6 (20%)	6 (20%)	NS
Duration of surgery (min)	79.6 (23.9)	83.6 (19.8)	0.487
Blood loss (mL)	78.2 (33.8)	60.0 (29.2)	0.030

Values are given as Mean ± SD, or number of patients (%).

**Table 2:** The degree of adverse reactions of hemostatic stuffing after FESS.

Groups	Level 1	Level 2	Level 3	*χ* ^2^	*P* value
Placebo group (*n*)	2	14	14	6.933	0.031
DEX group (*n*)	8	16	6		

**Table 3:** Quality of intraoperative surgical field during FESS.

Groups	Level 1	Level 2	Level 3	Level 4	Level 5	*χ* ^2^	*P* value
Placebo group (*n*)	3	11	14	2	0	8.019	0.046
DEX group (*n*)	5	18	4	1	0		

**Table 4:** The times of body movements during FESS.

Groups	0 times	1 times	2 times	3 times	4 times	*χ* ^2^	*P* value
Placebo group (*n*)	5	11	7	6	1	10.376	0.035
DEX group (*n*)	15	11	4	1	0		

**Table 5:** Surgeon and patient satisfaction scores.

	Placebo group (*n* = 30)	DEX group (*n* = 30)	*P* value
	Median (IQR)	Median (IQR)	
Surgeon satisfaction score (1–10)	7 (2)	8 (1.25)	0.000
Patient satisfaction score (1–10)	6 (1)	9 (1)	0.000

Values are given as Median (IQR (range)).
